# What do women with sexual interest in children tell us about the assumed cause of their sexual interest in children, (non-)disclosure, and professional help?—Results of a qualitative content analysis

**DOI:** 10.1038/s41443-023-00677-6

**Published:** 2023-03-06

**Authors:** Safiye Tozdan, Greta Hübener, Peer Briken, Johanna Schröder

**Affiliations:** 1 Institute for Sex Research, Sexual Medicine and Forensic Psychiatry, University Medical Center Hamburg-Eppendorf, Martinistraße 52, D-20246 Hamburg, Germany; 2 Institute for Clinical Psychology and Psychotherapy, Department of Psychology, Medical School Hamburg, Hamburg, Germany

**Keywords:** Health care, Medical research

## Abstract

Research on women with sexual interest in children is still rare, especially regarding women’s own theories about the cause of their sexual interest in children, their experiences with (non-)disclosure, and professional help. In the context of a broader online study, we provided 50 women with a sexual interest in children under the age of 14 years (mean age: 33.6, SD = 11.1) with open questions regarding their own theories about what causes their sexual interest in children, experiences with disclosure and non-disclosure, and experiences with and opinions about professional help. Analyses were conducted using an inductive qualitative content analysis method that aimed at ordering and structuring manifest and latent content by categorizing qualitative data. Results revealed that participants mainly think that past experiences caused their sexual interest in children (Σ = 16), e.g., abusive or non-abusive sexual experiences during childhood. Some participants think that their sexual interest in children is a disposition they were born with (Σ = 8). Disclose of sexual interest in children to another person was reported by 56.0% of the present sample and led to rather positive consequences (Σ = 24, e.g., acceptance or support). Those who did not disclose (44.0%) mainly did so due to fear of rejection and/or stigmatization (Σ = 24). A total of 30.0% already sought help due to their sexual interest in children and frequently reported negative experiences (Σ = 15). A frequent statement participants made on how to reach women with sexual interest in children in order to offer professional help was the destigmatization of sexual interest in children (Σ = 14). We recommend that women with sexual interest in children should be taken more seriously among research and in prevention measures.

## Introduction

In this study, the term “sexual interest in children” is abbreviated by “SIC” and includes pedophilic (regarding prepubescent children aged 10/11 years or younger; [1]) and hebephilic sexual interest (regarding pubescent children aged 11–13/14; ref. [2]). The term does not necessarily imply that diagnostic criteria are met. Paraphilic interests in females, such as pedophilic interest, have been neglected in research for a long time compared to paraphilic interests in males. In recent years, researchers focused on estimating the prevalence of SIC in females among non-clinical samples (e.g., [3–5]). Results revealed rates ranging from 0.4 to 9.6% and proving that a phenomenon like SIC does not only exist in men [3–5]. Most recently, Lievesley and Lapworth [6] conducted interviews with six women who had self-identified SIC. The authors applied a semi-structured interview covering the themes sexual attractions, methods and strategies used for managing sexual attractions, as well as experiences of disclosure and seeking support. Results underlined the uniqueness of the experience of being a woman with SIC, showed that the women felt socially isolated, and that they feared negative consequences when disclosing their SIC to others including health professionals [6]. Qualitative research on women with SIC is rare. Furthermore, as far as we know, subjective assumptions about possible reasons for SIC in women has not been investigated so far.

## The present study

In order to address the lack in current research mentioned above, we aimed at investigating women with SIC. We asked women with SIC an open question concerning their own theories about the subjective cause of their SIC. We consider it relevant to find out what women with SIC think why they developed a sexual attraction to children as it gives insights into how they perceive and understand their SIC. By this, future treatment programs that address women with SIC may be conceptualized more appropriate by taking into account different possible backgrounds of the women’s SIC. We further asked questions on the women’s experiences with disclosure, non-disclosure, and professional help. The questions were part of an online survey addressing women with SIC [7].

## Methods

### Procedure

Data was gathered via an online survey from July to December 2020. We collected data among German and English speaking participants. Firstly, we collected data among German participants via one study link generated with Qualtrics (www.qualtrics.com). In order to allow participants to conduct the survey via browsers guaranteeing a maximum of anonymity (e.g., “Tor Browser”), we also started collecting data among German participants via a study link generated with LimeSurvey (www.limesurvey.org). The English version of the study was also programed using LimeSurvey. All three study links were mainly spread on websites directed towards individuals with SIC.^1^ The German study links were also distributed via the Homepage of the research project, the Instagram account of the University Hospital Hamburg-Eppendorf, emails to the German psychotherapist’s chambers, as well as several other websites that were not explicitly directed to individuals with SIC.^2^ Additionally, the German study links were given to staff members of the outpatient treatment center of the Institute for Sex Research, Sexual Medicine and Forensic Psychiatry at the University Hospital Hamburg-Eppendorf and to a counseling center in Hamburg (“Wendepunkt e.V.”) for minors and young adults with sexually conspicuous behavior. Participants were informed that the survey is about SIC under the age of 14 years and is directed at women who are at least 18 years of age. Before beginning the survey, informed consent was obtained from all participants. The study was approved by the ethics committee of the Local Psychological Ethics Committee of the University Medical-Center Hamburg (reference number: LPEK-0110).

### Participants

Inclusion criteria for the present study were [1] being born as a female or identifying with the female gender, [2] being at least 18 years old, and [3] having a self-identified SIC under the age of 14 years. Exclusion criterion was identifying solely with the male gender. The total sample consists of 50 participants (40 German speaking, 10 English speaking) and constitutes a subsample of the participants reported by Tozdan et al. [7]. Table 1 shows sample characteristics. A total of 46 participants reported that they were female assigned at birth. Of those, 37 participants stated that they identify with the female gender, five indicated that they identify with both the male and the female gender, two indicated that they identify with another gender without further specification, and two indicated that they identify with no gender. Three participants were male assigned at birth. Of those, two participants stated identifying with the female gender and one indicated identifying with both male and female gender. One participant reported another gender at birth and reported identifying with both the male and the female gender. Thus, four participants did not fit the traditional definition of women as being born female. In the context of current public debates on gender, we decided to not exclude participants from our study who identify with the female gender. It might be assumed that these participants are not comparable to the others since the sex assigned at birth affects sexual arousal (e.g., [8]), even after the physical transformation in male-to-female transsexuals [9]. However, sample characteristics in the present study and previously results on this sample revealed no fundamental differences between participants who were born female and those who were born male and identify as female [7, 10]. Therefore, we assume that the inclusion of these four participants did not severely distort the overall results. One participant stated having a close “relationship” with a minor (aged 12 years). The 20 participants who reported having ever been diagnosed with a mental disorder specified depression (75.0%), anxiety disorders (35.0%), posttraumatic stress disorder (30.0%), personality disorders (25.0%), obsessive compulsive disorder (10.0%), bipolar disorder (5.0%), schizoaffective disorder (5.0%), autism (5.0%), dissociative disorder (5.0%), eating disorder (5.0%), attention deficit hyperactivity disorder (5.0%), attention deficit disorder (5.0%), Munchhausen by proxy syndrome incl. Munchhausen by adult proxy syndrome^3^ (5.0%), and substance abuse (5.0%).

**Table 1:** Sample characteristics for the total sample as well as for the subsamples “female assigned at birth” and “male/other assigned at birth”.

	Total sample (*n* = 50)	Female assigned at birth (*n* = 46)	Male/other assigned at birth (*n* = 4)
	M^a^(SD^b^)/N^c^(%^d^)	Md^e^/N(%)	Md/N(%)
Age at data collection	33.6 (11.1)	33.0 (11.1)	27.0 (12.3)
Education level			
Low	13 (26.0)	12 (26.1)	1 (15.0)
Moderate	9 (18.0)	8 (17.4)	1 (25.0)
High	28 (56.0)	26 (56.5)	2 (50.0)
Partnership status			
No	27 (54.0)	24 (52.2)	3 (75.0)
Yes	23 (46.0)	22 (47.8)	1 (25.0)
Partner’s age at data collection	32.9 (11.1)	33.5 (11.2)	23.0 (−)
Sexually attracted to (multiple answers possible)			
Male Infants (0–4 years)	19 (38.0)	18 (39.1)	1 (25.0)
Female Infants (0–4 years)	21 (42.0)	19 (41.3)	2 (50.0)
Boys before puberty (5–10 years)	25 (50.0)	23 (50.0)	2 (50.0)
Girls before puberty (5–10 years)	29 (58.0)	27 (58.7)	2 (50.0)
Boys at puberty (11–13 years)	22 (44.0)	20 (43.5)	2 (50.0)
Boys at puberty (11–13 years)	15 (30.0)	13 (28.3)	2 (50.0)
Ever been diagnosed with a mental disorder			
No	30 (60.0)	27 (58.7)	3 (75.0)
Yes	20 (40.0)	19 (41.3)	1 (15.0)

^a^Mean value.

^b^Standard deviation.

^c^Absolute share in the sample.

^d^Percentage share in the sample.

^e^Median.

### Measures

Sample characteristics were assessed. Participants were further asked whether they are in a steady relationship and if so, specified their partner’s age in years. Diagnosis of mental disorders was gathered by a free text field and the age groups they were attracted to by a multiple choice question. Participants were also asked which age groups they are sexually attracted to. For the purpose of the present study, we included five open questions that were presented during the online survey. Participants gave their answers using free text fields. For replicable results, we describe the open questions in detail below.

#### Cause of SIC

Participants were asked “What do you think is the reason why you developed a sexual interest in children?”.

#### (Non-)disclosure

Participants who indicated that they have ever spoken with someone in their social environment (e.g., friends, family) about their SIC were presented with the question “How did this person/these persons react to this?”. Participants who indicated that they have never spoken with someone in their social environment (e.g., friends, family) about their SIC were presented with the question “Why have you never spoken with someone in your social environment (e.g. friends, family) about your sexual interest in children?”.

#### Professional help

Participants who stated that they have ever sought professional help because of their SIC (e.g., at information centers, from physicians, or therapists) were presented with the question “What was your experience with this?”. Finally, all participants were asked “In your opinion, what is the best way to reach affected women if they need professional help?”

### Data analysis and presentation

Quantitative data analyses for the sample characteristics were conducted using IBM SPSS Statistics, version 22 [11]. For continuous variables mean values and standard deviations were reported for the total sample. For the two subsamples medians were reported as the subsample “male/other assigned at birth” only included four participants.

The qualitative data was analyzed in accordance to qualitative content analysis by Mayring [12] that aimed at ordering and structuring manifest and latent content by categorizing qualitative data. An inductive (also called conventional) approach was applied which means that categories are generated based on the study material. A deductive approach implies a previously prepared category system that is grounded on a theory or empirical research [12]. As there is not enough qualitative research on women with SIC, an inductive approach was considered appropriate. Content analysis was conducted in order to use a descriptive approach in both coding of the data and its interpretation of quantitative counts of the codes. In a first step, the second author (female, medical student) worked through the data and attempted to identify relevant themes in participants’ responses. The themes were ordered and superordinate categories were formed. In a second step, the first author (female, post-doc psychologist) applied the preliminary category system to the data in order to prove and modify it (e.g., verifying, abstracting, and reducing categories). No software was used to manage the data. For reflection on and discussion about the coding process, institutional meetings with colleagues were held. The “Consolidated criteria for reporting qualitative studies (COREQ): 32-item checklist” [13], which was designed for qualitative research approaches, was completed and attached as supplementary file. As we conducted a quantitative online survey including few open questions, not all items were applicable.

## Results and discussion

### Subjective causes of SIC

A total of 86.0% answered the question on the assumed cause of their SIC. Table 2 shows all response categories and how often they occurred. The largest category consists of assumed causes that lie in the participants’ own childhood or at least in their past. For example, the answer

“Because of my own trauma history.”

**Table 2:** Response categories for the subjective cause of SIC in female participants (*n* = 43).

	Σ	
Past experiences	16	
Own victimization		7
Past sexual experiences^a^		5
Other past experiences^b^		4
Attitudes towards children	13	
Characteristics of children/adolescents		9
Emotional affiliation with children/the childish world		3
Disposition	8	
General sexual desire	4	
External pressure	1	

One participant can occur in multiple response categories.

*SIC* sexual interest in children.

^a^Not explicitly abusive.

^b^Not explicitly sexual or abusive.

was categorized as *own victimization*. This subjective estimation adds to environmental theories among researchers claiming that SIC develops in the course of a social learning process (e.g., [14]). It is assumed that individuals who were sexually abused during their childhood become sexually attracted to children during adulthood and may have the desire to sexually offend against children because they repeat the abuse they experienced as children. At least, self-experienced sexual abuse is more common among men with SIC than among others (e.g., [15]). Some participants in the present study reported non-abusive sexual experiences as the reason for their SIC. For example, one participant referred to her *past sexual experiences* with her younger brother that helped both of them to survive sexual abuse:

“With my little brother I had a wonderful innocent tendered love and sexual relationship that helped us and strengthened us for the world to survive against sexual abuse, school anxiety, bullying…”

Another participant described jointly masturbation with her older sister as the reason for her SIC stating that this experience is the most intense and most positive memory of her childhood. She further explained that a few years later she did the same with her younger cousin and then with the little sister of a friend. She was sexually aroused by the fact that she acted as a kind of teacher showing the younger girls how their body works. Finally she stated:

“…that pattern remained although I grew older. You cannot tell a 20-year old how their body functions.”

These responses are in line with considerations grounded on learning theories assuming that during first sexual experiences with children of the same age there might be a conditioned coupling of previously neutral stimuli (e.g., a child’s body schema) with unconditioned sexual satisfaction which causes a SIC [16]. Four participants named *other past experiences* that were not explicitly sexual or abusive, e.g.:

“Past” or

“An early puberty triggered by steroids from asthma breathing treatments.”

Nine participants referred to *characteristics of children/adolescents* as the cause for their SIC, e.g.:

“… I found boys/young men beautiful”

or described an emotional affiliation with children/the childish world, e.g.:

“… I envy the beautiful way that children see the world without sexuality clouding their vision. I hate the adult world for trying to pervert them. I want to be a child again,…”

Such features remind of the concept of emotional congruence with children which is associated with an exaggerated cognitive and emotional affiliation with childhood, child-like characteristics, strong non-sexual liking of children, and positive views of children and childhood [17]. It has been shown that emotional congruence with children is related to pedophilic interest and sexual recidivism risk among men who sexually offended against children [18]. Future research should find out to what degree the concept of emotional congruence with children is also important in women with SIC. Some participants specified that their SIC is a *disposition*, e.g.:

“I was born with it.”

which fits the assumption that a genetic component has an influence on the development of sexual interest and masturbation fantasies. In fact, Alanko et al. [19] reported that among participants with a SIC a non-additive genetic component could explain 14.6% of the total variance in the data. In contrast, environmental influences that were not shared by the twins explained 85.4% of the total variance indicating that the environment has a greater influence on SIC then genes [19].

Four participants reported that their *general sexual desire* is the reason for their SIC, e.g.:

“Because I like sex and because it is beautiful/awesome to me.”

Research has shown that a sexual interest in children can occur in the context of compulsive sexual behavior [20]. Thus, these participants may not have a sexual interest in children in terms of pedophilia but rather might show indicators for sexual preoccupation or compulsive sexual behavior. Finally, one woman stated that *external pressure* caused her SIC:

“I was persuaded by my boyfriend at the beginning. But it was good for me and then I was attracted to it.”

Some participants of the present study mentioned more than one reason for their SIC, e.g.:

“I think that the major cause is disposition… Eventually the emotional abuse and mental violence during my childhood contributed to the anchoring of the preference during puberty because I felt emotionally closer to children and I partly still do today.”

This participant reported both a *disposition* and *past experiences* causing her SIC and additionally addressed an *emotional affiliation with children/the childish world*.

Ten participants answered that they had no idea why they have a SIC.

### Experiences with disclosure

A total of 56.0% disclosed to another person and answered the question on the reactions to their disclosure (Table 3). Most participants reported *positive reactions* after disclosing. Both results are in line with those by Wagner et al. [21] who reported that about half of their male sample directly disclosed to another person which was rather associated with positive consequences, such as social acceptance. On the one hand, it might be that people rather react positive to a known person who admits to be sexually interest in children. This would imply that individuals with SIC should be encouraged to open up to the people around them in order to overcome the feeling of isolation. On the other hand, and more likely, individuals with SIC may anticipate how the people around them will react and obviously rather disclose to people whose reaction are expectedly positive. One participant reported *unspecified* positive reactions and *support*:

“Reactions have been uniformly positive and supportive. I am careful about who I confide in.”

**Table 3:** Response categories for the reactions to disclosure of SIC of the female participants (*n* = 28).

	Σ	
Positive reactions	24	
Unspecified		6
Acceptance		6
Support		5
Other person agreed with the SIC		4
Understanding		3
Negative reactions	7	
Disbelief		3
Contact termination		2
Shame and disgust		1
Unspecified		1
Neutral reactions	5	
Search for information	2	
Need for communication with others	1	

One participant can occur in multiple response categories.

*SIC* sexual interest in children.

This response not only shows that the women made positive experiences with her disclosure but also reflects that she saw the need of being aware who she tells about her SIC. As society overestimates the relation between SIC and sexual offending against children, at least, men with SIC are generally seen as dangerous [22]. The stereotype of dangerousness is strongly connected to desires to punish or to avoid the men with SIC. As a consequence, men with SIC fear the disclosure of their SIC and make efforts to cover it up since they are afraid of negative social consequences [22]. Although these results need to be validated in female samples, it can be assumed that women with SIC also anticipate negative reactions and thus decide to not disclose. Some participants mentioned that they experienced acceptance by the other persons, e.g.:

“…They accept me regardless.”

or stated that they were supported by the people around them, e.g.:

“She has been supportive.

Four participants reported that the *other person agreed with the SIC*:

“My partner is pedophilic himself and therefore reacted calmly.”

Three participants claimed that the person reacted by understanding, e.g.:

“Showed a lot of understanding.”

Few participants mentioned *negative reactions* of the other person including *disbelief*, e.g.:

“Restrained, I was only taken seriously to a limited degree. I have a husband and an active sex life. It has to be imagination.”

It can be assumed that people generally think that SIC is a male phenomenon. Thus, when a women mentions having a SIC people probably tend to minimize that statement and do not take the women seriously. The six women with SIC that were interviewed by Lievesley and Lapworth [6] claimed that women with SIC are a ‘minority within a minority’. They felt overlooked in social discussions about SIC as people do not recognize that women with SIC exist [6]. Furthermore, as far as we know so far it seems to be common for women with SIC to have an adult partner and a sex life as most women with SIC from the current sample also reported a sexual interest in adults [7]. Thereby their SIC mays not be detectable from the outside.

Two participants stated that the person they disclosed to responded by *contact termination*, e.g.:

“Frightened in the first instance—she sealed herself off and did not want to have contact for a while (3 month)…“,

And one answer included *shame and disgust*:

“Mother cried and was speechless. Asked many questions. Said she would feel shame and disgust…”

This woman further revealed that her mother later *searched for information* on the internet regarding a prevention program where the woman participated. Her brother expressed his *need for communication with another person*:

“…Brother …asked me for permission to speak with a trusted person.”

One participant mentioned an unspecified negative reaction:

“Very negative”.

Finally, five participants mentioned neutral reactions, e.g.:

“Predominantly positive or neutral.”

At least for men, research has shown that negative social consequences after disclosing that one is sexually attracted to children, such as social ostracism, can lead to more emotional and social problems which may in turn even increase the risk of offending against children [23]. The participant mentioned above who experienced contact termination for three months further described that later she was able to explain everything to the person and that they became friends. However, the narrative indicates that she experienced the three month of non-contact as negative before she became friend with the other person. It appears to be relevant to investigate the long-term effects of negative consequences after disclosing that one has a SIC among females.

### Reasons for non-disclosure

A total of 44.0% of participants stated that they did not disclose to anyone mainly due to the fear of certain consequences (Table 4). This confirms research on stigma in men with SIC mentioned above (e.g., [22]). One participant described her *fear of rejection* of her best friend:

“I could never talk about it to anyone, not even to my best friend, because I have great fear that she finds me repulsive or that I cannot see her anymore.”

**Table 4:** Response categories for the reasons for non-disclosure (*n* = 22).

	Σ	
Fear	24	
Fear of rejection		9
Fear of stigmatization		6
Fear of other negative consequences		5
Unspecified fear		4
Taboo	3	
Shame	2	
No need	2	

One participant can occur in multiple response categories.

Another participant specified her *fear of rejection* and her *fear of stigmatization* and *other negative consequences* as follows:

“Fear of getting stigmatized and ostracized; and of police investigations, although I have never done anything. It could harm my family when others know. I do not want to be bullied or to get physically attacked.”

Some participants referred to *unspecified fears*, e.g.:

“Fear”

Similar to our participants, the women examined by Lievesley and Lapworth [6] indicated that they perceive risks associated with disclosure of their SIC. The authors refer to the possible consequences of internalizing a social stigma that can be observed in sexual minority adults including depression, anxiety, substance abuse, and suicidality (e.g., [24]). It can be presumed that such consequences may be also relevant for women with SIC. In line, 40.0% of our participants reported that they have been diagnosed with a mental illness and mainly specified that it was the diagnosis depression.

Of course, non-disclosure can protect affected individuals from stigma and discrimination. However, concealing can also make it difficult to find social support [25] and may lead to isolation and loneliness in women with SIC [6]. Therefore, women with SIC should be encouraged to disclose to persons they trust and/or to professionals as most women in the present study experienced positive consequences from doing so. Nevertheless, our results also demonstrated that there are risks associated with the disclosure of SIC. Additionally, research has proven that people in general wish to punish or to avoid men with SIC [22]. Ultimately, there probably is no general rule in terms of disclosure of SIC. Affected women need to make an individual decision and at best, they are supported by professionals who have experience in treating individuals with SIC. Three participants further mentioned *taboo* as the reason why they do not disclose, e.g.;

“Extreme taboo”

Two participants named *shame* as the reason for their non-disclosure which in one case seems to be related to the stigmatization of SIC:

“I feel ashamed to admit this to a trusted person. After all, it is a delicate and stigmatized topic.”

Another participant claimed *no need* for disclosure of her SIC by writing:

„ I don’t want to, it’s private, like my sexuality (bisexual)…”

She further explained that a disclosure would have negative consequences:

“…And I know it would be very dangerous, and they would never accept it.”

which indicates that her shame may be based on her *fear of other negative consequences*.

Two participants indicated that they had no idea why they do not disclose to another person.

### Experiences with professional help

A total of 30.0% of participants reported having sought help due to their SIC and most *experiences* with professional help appear to be *negative* (Table 5). This result is in line with research showing men with SIC mostly report negative consequences (e.g., inappropriate treatment methods and stigmatization) when they disclose to general professionals in the health care system [21]. Five participants in the present study reported that the professionals were *not helpful*, e.g.:

“No help for coping.”

**Table 5:** Response categories for experiences with professional help (*n* = 15).

	Σ	
Negative experiences	15	
Not helpful		5
Disbelief		4
Rejection		3
No courage/no trust towards professional		2
Disgust		1
Positive experiences	7	
No judgment		2
Helpful		2
Genuine interest and empathy		1
Not being alone		1
Unspecified		1
Neutral experiences	1	

One participant can occur in multiple response categories.

Some participants described that the professionals’ reaction included *disbelief*, e.g.:

“They try to convince me it’s a phase.”

This result indicates that there are professionals in the health care system who think that SIC cannot exist in females. Obviously there has to be more information among professionals regarding SIC in women. Although SIC does not necessarily lead to child sexual abuse, research on male samples demonstrated that SIC contributes to child sexual abuse [26] and has been proven to be one of the most important risk factors for sexual recidivism among men [27]. As it cannot be assumed that this male-based finding applies to women, research to that effect is needed. Two participants stated that they had *no courage/no trust in professionals*, e.g.:

“…Trying to start therapy again but it’s so difficult to trust again. I just want help and to make good choices…”

One participant also mentioned that she felt her therapist was *disgusted*:

“My outpatient therapist was not a big help to me. She condoned the topic but we hardly talked about it. During the last session, I felt like I could grab her disgust.”

In another context she even experienced *rejection* by the health care system:

“When I brought up the topic during a hospital stay, they had a team meeting discussing whether it is still possible that I was further treated on that ward.”

This outlines how important it is that general professionals in the health care system are well educated concerning SIC. As they are part of the society they may also believe that people with SIC automatically conduct child sexual abuse and cannot imagine that part of them decide to not act on their SIC. However, the same participant also reported *positive experiences* with professionals in other contexts:

„The psychologist at my last hospital stay was very calm and never seemed to be disgusted or emotional; I could talk about it to her without having the feeling to be judged.”

This participant highlighted that she did felt *no judgment* due to her SIC indicating that she felt accepted. Since SIC is a highly stigmatized topic (e.g., [22]), it seems plausible that the feeling of being accepted and not judged may be of high importance when treating women with SIC. Compared to men, it might be even more relevant for women to feel accepted given their greater need for affiliation [28]. Two women reported that their experiences were *helpful*, e.g.:

“…Therapist was not specialized in this area, but could give me some helpful exercises I can apply in everyday life…”

The same participant stated that her therapist shows *genuine interest and empathy*. A further participant mentioned:

“Good: I am not alone.”

This quote indicates that the good experience of this participant was that she felt not alone. Similar to acceptance, the feeling of *not being alone* may be highly relevant in the treatment of women with SIC as they possibly carry a sense of isolation and loneliness [6]. One participant reported *unspecified* positive experiences after a period of *disbelief*:

“Negative experiences at the beginning since I was not taken seriously because I was so young. Afterwards positive experiences, but then I was older.”

Finally, one participant claimed *neutral experiences*:

“Therapists predominantly reacted neutral…”

We consider the consequences these experiences—either positive, nor negative, nor neutral – may have on the individual course of women with SIC to be relevant. It seems at least reasonable that the women in the present study who made negative experiences possibly tend to avoid further contacts to professionals regarding their SIC. Positive experiences on the other hand may encourage women with SIC to stay in treatment which might be a protective factor against child sexual abuse. Future research should address the question which impact such experiences can have on women with SIC and on their possible risk to sexually abuse children.

### Suggestions for how to reach women with SIC

A total of 78.0% of participants answered the question on how to reach women with SIC who are in need of professional help (Table 6) indicating that the stigmatization of individuals with SIC appears to be the biggest barrier for reaching women with SIC. In addition to the *destigmatization*, some also mentioned the *assurance of anonymity* as important, e.g.:

“Internet, anonymously, without stigmatization and fear of punishment.”

**Table 6:** Response categories for the question on how to reach women with SIC to offer help (*n* = 39).

	Σ	
Acceptance	19	
Destigmatization of SIC^a^		14
Awareness that women with SIC exist		4
Self-acceptance		1
Media/advertising	8	
Treatment features	8	
Assurance of anonymity		3
Special treatment program for women with SIC		3
Supportive therapists		1
No enforcement to sex with adults		1
Legalization of sexual contacts with children	5	
Not possible	3	

One participant can occur in multiple response categories.

*SIC* sexual interest in children.

^a^This category also includes answers that refer to the social acceptability of SIC as well as to not being judged or punished.

Based on our results, it appears that women with SIC struggle with the same problems as men with SIC regarding stigmatization [22] and fear of negative consequences [21]. Some participants also stated that there need to be more *aware that women with SIC exist*, e.g.:

„First of all there is a need for more enlightenment regarding this topic. In people’s minds men are still the ones that have a sexual interest in children/adolescents…”

She further explained that females might already be reached at school when they are adolescents who eventually develop a SIC. The students should be enlightened about prevention programs so that they know where to find help in the future. The participant added that *special treatment programs for women with SIC* are necessary:

„…An additional program would make sense addressing women. I think men and women should not be mixed up here.”

One participant stated that *self-acceptance* is needed:

“…The most important step is to be honest with you and to accept yourself, so that you can talk about it and work on it…”

Eight women suggested using *media and advertising* to reach out for women with SIC, e.g.:

“…flyers at pediatricians?…”

or

“Raise awareness through TV and internet ads…”

Our results show that women with SIC appear to be aware of the social stigma that surrounds pedophilia. Many females in the present study clearly mentioned the need for destigmatization of people with SIC. They considered a feeling of acceptance relevant in order to reach women with SIC to offer help and partly highlighted that special treatment program for women should be implemented. It seems to be necessary to raise awareness for this topic among the general public and targeted professional groups. Regarding treatment features, one participant underlined the importance of *supportive therapists* referring again to the negative experiences she made during her last treatment*:*

“…I wish therapists understood that the best thing for everyone is to actually be supportive instead of abusive. I’m seriously hurting about this last therapist I trusted.”

And one participant spoke out against the *enforcement to sex with adults*:

“Don’t encourage us to have sex with adults to change things bc it is traumatic for us and makes us feel like we are being raped.”

At least five participants claimed that the *legalization of sexual contacts with children* would help women with SIC, e.g.:

„Tenderness and consensual sex and motherly love should be decriminalize, that would be better for all and a better protection for children of exploitative and violent sexual relationships.”

This participant described her relationship with her daughter as more intimate then the relationship to her husband. It might be assumed that she conduct sexual acts with her daughter as she seems to be convinced that “consensual sex and motherly love” with children is supposed to protect children from sexual violence. Some research results indicate that cognitive distortions found in men regarding sexual acts with children also are relevant in women who sexually offended against children. For example, Beech et al. [29] analyzed interviews among 15 women who were incarcerated for child sexual abuse. The authors found that implicit theories underlying men’s cognitions who sexually offended against children for the most part could be identified in women. One of these implicit theories implies seeing children as sexual objects and being sexually attracted to or aroused by the abused children. The abused children are considered to enjoy the sexual offending behavior and actively seek to repeat the experiences [29]. Other research results indicate that women who sexually offend against children are less likely than men to be characterized by motivations and cognitions associated with the sexualization of children (e.g., [30]). Cognitive distortions have been demonstrated to be a risk factor for recidivism in men who sexually abused children [31]. Further research is needed to determine whether this male-based finding applies to women. Three participants mentioned that reaching out for women with SIC is *not possible*, e.g.:

“…Unless a pedo reaches out to you, there’s no way you can know and reach out to them…”

Nine participants specified that they had no idea how to reach women with SIC.

### Limitations

The major limitation of the present study is that the sample is small and not representative. Moreover, ten participants entered the study via the English study link. The English version of the study was translated by someone whose first language is English from a translation company. The translation and cultural adaptation of a questionnaire should include a forward-backward translation by qualified translators as well as subsequent validation and harmonization processes. In addition, one participant entered the survey via a German study link, but appears to be not German-speaking. Free text fields showed that this participant answered in English. However, the response pattern of this participant was fully comprehensible and consistent. We were additionally not able to identify and exclude females with obsessive–compulsive disorder including obsessions related to children that do not indicate a SIC in the sense of pedophilia [32]. At least, two participants reported that they were given the diagnosis obsessive compulsive behavior during their life time. Finally, it has to be mentioned that the present results were partly discussed referring to findings on men with SIC or men who sexually abused children that were not validated on female samples. As previous research revealed that there are remarkable differences between men and women in terms of sexuality (e.g., [8]), findings in men are not necessarily transferable to women regarding SIC. In summary, the validity and generalizability of the present results are therefore limited.

## Conclusion

Women with SIC need to be taken more seriously among research as well as in prevention measures. Prevention programs should address females with SIC more explicitly. In addition, awareness of SIC in females among society should be promoted as well as the fact that SIC is not equal to child sexual abuse to counteract stigmatization of people with SIC. Feelings of exclusion and stigmatization in women with SIC might be compensated during treatment which may decrease the risk to sexually offend against children. It may also be important to ask women with SIC about their own theories on the cause of their SIC in order to discover own abusive childhood experiences. Women with SIC should be supported regarding the disclosure of their SIC. Possible negative consequences following disclosure need to be addressed during treatment.

## Supplementary information

Supplementary File_ Consolidated criteria for reporting qualitative studies (COREQ).docx

aj-checklist.pdf

## Data Availability

The data that support the findings of this study are available from the corresponding author, ST, upon reasonable request.

## Footnotes

1 www.gsa-forum.de, www.kinder-im-herzen.de, www.schicksal-und-herausforderung.de, www.paedoseite.home.blog, www.krumme13.org, www.virped.org.

2 www.gute-frage.net, www.paradisi.de, www.psychologie.gofeminin.de, www.urbia.de.

3 The actual diagnosis would be factitious disorder imposed on another.
