# Supplementary material for: What do women with sexual interest in children tell us about the assumed cause of their sexual interest in children, (non-)disclosure, and professional help?—Results of a qualitative content analysis
Source: Int J Impot Res. 2023 Mar 6;37(3):215–23. doi: 10.1038/s41443-023-00677-6 (PMC11981925; doi:10.1038/s41443-023-00677-6)
Supplement: Supplementary file 1 — Supplementary File_ Consolidated criteria for reporting qualitative studies (COREQ).docx [file 41443_2023_677_MOESM1_ESM.docx]

Consolidated criteria for reporting qualitative studies (COREQ): 32-item checklist

| **No Item** | **Guide questions/description** | **Application** |
| --- | --- | --- |
| ***Domain 1: Research team and reflexivity*** | | |
| Personal Characteristics | | |
| 1. Interviewer/facilitator | Which author/s conducted the interview or focus group? | Not applicable |
| 2. Credentials | What were the researcher’s credentials? E.g. PhD, MD | X |
| 3. Occupation | What was their occupation at the time of the study? | X |
| 4. Gender | Was the researcher male or female? | X |
| 5. Experience and training | What experience or training did the researcher have? | X |
| Relationship with participants | | |
| 6. Relationship established | Was a relationship established prior to study commencement? | Not applicable |
| 7. Participant knowledge of the interviewer | What did the participants know about the researcher? e.g. personal goals, reasons for doing the  research | Not applicable |
| 8. Interviewer characteristics | What characteristics were reported about the interviewer/facilitator? e.g. Bias, assumptions,  reasons and interests in the research topic | Not applicable |
| ***Domain 2: study design*** | | |
| Theoretical framework | | |
| 9. Methodological orientation and  Theory | What methodological orientation was stated to underpin the study? e.g. grounded theory,  discourse analysis, ethnography, phenomenology, content analysis | Not applicable |
| Participant selection | | |
| 10. Sampling | How were participants selected? e.g. purposive, convenience, consecutive, snowball | X |
| 11. Method of approach | How were participants approached? e.g. face-to-face, telephone, mail, email | X |
| 12. Sample size | How many participants were in the study? | X |
| 13. Non-participation | How many people refused to participate or dropped out? Reasons? | Not applicable |
| Setting | | |
| 14. Setting of data collection | Where was the data collected? e.g. home, clinic, workplace | X |
| 15. Presence of non-participants | Was anyone else present besides the participants and researchers? | Not applicable |
| 16. Description of sample | What are the important characteristics of the sample? e.g. demographic data, date | X |
| Data collection | | |
| 17. Interview guide | Were questions, prompts, guides provided by the authors? Was it pilot tested? | Not applicable |
| 18. Repeat interviews | Were repeat interviews carried out? If yes, how many? | Not applicable |
| 19. Audio/visual recording | Did the research use audio or visual recording to collect the data? | Not applicable |
| 20. Field notes | Were field notes made during and/or after the interview or focus group? | Not applicable |
| 21. Duration | What was the duration of the interviews or focus group? | Not applicable |
| 22. Data saturation | Was data saturation discussed? | Not applicable |
| 23. Transcripts returned | Were transcripts returned to participants for comment and/or correction? | Not applicable |
| ***Domain 3: analysis and findings*** | | |
| Data analysis | | |
| 24. Number of data coders | How many data coders coded the data? | X |
| 25. Description of the coding tree | Did authors provide a description of the coding tree? | X |
| 26. Derivation of themes | Were themes identified in advance or derived from the data? | X |
| 27. Software | What software, if applicable, was used to manage the data? | X |
| 28. Participant checking | Did participants provide feedback on the findings? | Not applicable |
| Reporting | | |
| 29. Quotations presented | Were participant quotations presented to illustrate the themes / findings? Was each  quotation identified? e.g. participant number | X |
| 30. Data and findings consistent | Was there consistency between the data presented and the findings? | X |
| 31. Clarity of major themes | Were major themes clearly presented in the findings? | X |
| 32. Clarity of minor themes | Is there a description of diverse cases or discussion of minor themes? | X |
